# Effects of educational animations using message framing on the appropriate antibiotic use by parents: a randomised, three-armed intervention study

**DOI:** 10.1186/s12889-025-23577-4

**Published:** 2025-07-03

**Authors:** Hitomi Kawamura, Hidehiko Sakurai, Takuya Sakamoto, Hiroto Arai, Kazuyuki Naoi, Rie Tsukui, Sanju Iwamoto, Madoka Aizawa, Keiko Kishimoto

**Affiliations:** 1 Department of Social Pharmacy, Graduate School of Pharmacy, Showa Medical University, 1-5-8 Hatanodai, Shinagawa-Ku, Tokyo, 142-8555 Japan; 2 Department of Pharmacy Administration, Faculty of Pharmaceutical Sciences, Hokkaido University of Science, 15-4-1, Maeda 7-Jo, Teine-Ku, Sapporo-Shi, Hokkaido, 006-8585 Japan; 3 Department of Pharmacy, Japan Organization of Occupational Health and Safety Tokyo Rosai Hospital, 4-13-21 Omoriminami, Ota-Ku, Tokyo, 143-0013 Japan; 4 Department of Pharmacy, Japan Organization of Occupational Health and Safety Kanto Rosai Hospital, 1-1 Kizukisumiyoshicho, Nakahara-Ku, Kawasaki-Shi, Kanagawa, 211-8510 Japan; 5 Department of Pediatrics, Japan Organization of Occupational Health and Safety Tokyo Rosai Hospital, 4-13-21 Omoriminami, Ota-Ku, Tokyo, 143-0013 Japan; 6 Rokugo Kodomo Clinic, 4-19-2 Nakarokugo, Ota-Ku, Tokyo, 144-0055 Japan; 7 Yamato Ikiiki Clinic, 2-5-12Yamato-Shi, Kanagawa , Yamatohigashi, 242-0017 Japan; 8 Kotokoto Clinic, 5-7-3Shinagawa-Ku, Tokyo , Nakanobu, 142-0053 Japan

**Keywords:** Antibiotics, Antimicrobial resistance, Parent, Educational animation, Behavioural economics, Message framing

## Abstract

**Background:**

Appropriate antimicrobial use should be encouraged among parents to prevent the increase in antimicrobial resistance. In Japan, antibiotics are widely used in paediatrics. This study aimed to create educational animations to educate parents on the appropriate use of antibiotics and to examine the effects of different animation scenarios using message framing on the intention to use antibiotics appropriately (hereinafter referred to as ‘intention’).

**Methods:**

In this intervention study, three educational animations were created: Animation A with a general message for Group A, Animation B with a gain-framing message for Group B, and Animation C with a loss-framing message for Group C. A questionnaire survey was conducted for parents of children who visited a medical facility before and after watching one of the animations. The primary questions focused on the intention to use antimicrobials appropriately and the assessment items based on the PMT that influence this intention (severity, vulnerability, response efficacy, self-efficacy, intrinsic rewards, and response costs).

**Results:**

Responses were obtained from 27, 29, and 31 participants in groups A, B, and C, respectively. Intention scores increased significantly in groups A (*p* = 0.001) and C (*p* < 0.001), whereas no significant difference was observed in group B (*p* = 0.237). Effect sizes were large in groups A (*r* = 0.62) and C (*r* = 0.64) and small in group B (*r* = 0.22). Regarding the PMT assessment items, severity significantly increased in groups A (*p* < 0.001) and C (*p* = 0.001), whereas no significant difference was observed in group B (*p* = 0.589). Effect sizes were large in groups A (*r* = 0.76) and C (*r* = 0.60).

**Conclusions:**

The addition of loss-framing messages to general knowledge was found to be more effective than that of gain-framing messages in improving intention. Positive expressions might reduce awareness of the severity of the risk of antimicrobial resistance and not improve intention. The use of loss-framing animation may be an effective tool for educating parents on the appropriate use of antibiotics.

**Trial registration:**

Japan Registry of Clinical Trials (Registration number: jRCT1030210605; Registration date: 10 February 2022).

**Supplementary Information:**

The online version contains supplementary material available at 10.1186/s12889-025-23577-4.

## Background

Antimicrobial resistance poses a global public health problem. Without intervention, the number of deaths from antimicrobial resistance is expected to reach 10 million worldwide by 2050, exceeding that from cancer [1]. A survey conducted in 2019 estimated that bacterial drug resistance was directly responsible for 1.27 million deaths worldwide and contributed to 4.95 million deaths [2]. In Japan alone, over 8,000 deaths due to methicillin-resistant *Staphylococcus aureus* and fluoroquinolone-resistant *Escherichia coli* were reported in 2017 [3]. The appropriate use of antibiotics is important in preventing the increase in antimicrobial resistance. The World Health Organization is coordinating a global campaign that includes the general public to raise awareness of antimicrobial resistance and prevent its further emergence and spread [4]. The National Action Plan on Antimicrobial Resistance [5], published in 2023, highlights the promotion of public awareness and educational activities regarding the knowledge of antibiotics and antimicrobial resistance. Oral antimicrobials account for 90% of the antibiotics used in Japan and are frequently used in paediatric patients [6]. Since 2018, paediatricians have received an ‘additional support for appropriate use of paediatric antimicrobial agents fee’ if they educate parents on the unnecessity of antibiotics for acute respiratory tract infection and acute diarrhoea treatment and do not prescribe antibiotics. Notably, the frequency of antibiotic prescriptions tends to be lower in facilities that receive the fee [7]. However, a 2023 survey of 222 parents of preschoolers in Japan found that 44.4% of parents would like their children to take antibiotics if they caught a cold [8]. Parents’ desire for and the inappropriate use of unnecessary antibiotics have been reported both nationally and internationally [9–11]. In a meta-analysis, factors leading parents to seek antibiotics for their children with upper respiratory tract infections (URTIs) included excessive worrying, poor knowledge about URTIs, lack of antibiotic awareness, and paucity of time and patience [12]. In our previous survey, Japanese mothers unconvinced about antibiotics not being prescribed present high expectations and misconceptions that antibiotic use will quickly improve symptoms of upper respiratory infections [13]; moreover, their parental concern and positive experiences with the use of antibiotics in the past contribute to this conviction [13]. It has also been reported that paediatricians are more likely to prescribe antibiotics inappropriately when they perceive parental expectations for such treatment [14].

Various measures have been implemented to improve antibiotic use by parents [11, 15–18].

When raising awareness, the suggestion that antibiotics are ineffective against viruses does not influence parents’ beliefs; in contrast, providing a clear explanation of when antibiotics are needed can prevent misunderstandings [17]. Reportedly, the use of fear-inducing and empowerment messages about antimicrobial resistance decreased antibiotic demand in adults [19]; however, parents did not change their likelihood of antibiotic use in children.

Although the Antimicrobial Resistance Clinical Reference Center in Japan has developed various awareness materials for public viewing [20], the types of messages and materials needed for more effective dissemination and education remain unclear. We believe that awareness messages and materials should help doctors with their explanations because there is limited time for them to provide patients with explanations in an outpatient setting.

One way to improve the effectiveness World Health Organization’s antibiotic awareness campaign is to use message framing in the delivery strategy [21], which has been researched in a variety of health behaviour fields [22–24]. A meta-analysis of the effects of message framing on health behaviours suggests that there are limits to the consistency of the hypothesis that framing effects vary by health behaviour role, indicating that the impact of situational and individual characteristics must also be considered [25]. Currently, no comparative studies have been conducted to determine more effective framing messages for the behavioural impact of appropriate antibiotic use.

Notably, in message-framing interventions, the most meaningful results are actual behavioural changes [25]. Conversely, a meta-analysis of the research on protection motivation theory (PMT) [26, 27], a health psychology theory introduced to motivate health behaviour, has found a correlation between protective motivation and future behaviour. According to PMT, protection motivation (behavioural intention) is aroused by two processes: threat appraisal, which consists of severity, vulnerability, and intrinsic and extrinsic rewards, and coping appraisal, which is constituted of response efficacy, self-efficacy, and response costs, potentially affecting preventative behaviour [28]. It is speculated that if the threat and coping appraisals are high, the protective motivation will be high; conversely, if they are low, protective motivation will be low as well [29].

Here, we created educational animations that added gain and loss-framing messages to general knowledge of antibiotics and examined the effects of different message delivery methods on parents' intention to use antibiotics appropriately and on the PMT components.

## Methods

This study implemented a randomised, three-armed intervention study design. It adhered to the principles outlined in the Declaration of Helsinki and received approval from the Showa Medical University (Tokyo, Japan) Research Ethics Review Board (approval number: 22–016-A). The study was divided into two components: creation of educational animations and evaluation of their effectiveness. Participants in the evaluation phase comprised parents who accompanied their children to a healthcare facility, were at least 20 years old, and had provided consent to participate in the study. The exclusion criteria included individuals who were healthcare providers for themselves or a family member, as well as those who had previously participated in the study. This study was reported in accordance with the CONSORT guidelines.

### Creation of educational animations

Animated videos were created as educational material for parents regarding the appropriate use of antibiotics, and the videos were viewed by parents and their children. Three animation scenarios were created: animations A, B, and C. Using materials from the Antimicrobial Resistance Clinical Reference Center, Animation A was designed to impart general knowledge about the mechanism of action of antibiotics, emphasising that antibiotics are ineffective against viral infections, the primary cause of upper respiratory infection and diarrheal symptoms, and highlighting the risks of antimicrobial resistance development due to antibiotic use. Animations B and C were developed by integrating gain- and loss-framing messages into Animation A, respectively. The gain-framing message stated that antimicrobial resistance can be inhibited by avoiding the unnecessary use of antibiotics. This would diminish the incidence of antimicrobial resistance-induced infections, thereby enabling the effective use of antibiotics in treating infections in children. In contrast, the loss-framing message was that the unnecessary use of antibiotics increases antimicrobial resistance. Accordingly, the message communicated that antibiotics were ineffective in treating antimicrobial-resistant infections in children and did not improve their medical condition. Participants in groups A, B, and C watched animations A, B, and C, respectively. The animations were silent and subtitled in Japanese for viewing in the waiting rooms of healthcare facilities and pharmacies. The playback time of each animation was set at approximately 1 min. The authors created character designs and storyboards, and an advertisement production company illustrated a character and edited animations based on these designs. At the end of all animations, we recorded actions by parents to prevent antimicrobial resistance, such as ‘Do not stop antibiotics at your own discretion, but take them as prescribed’.

### Measuring the effectiveness of educational animations

An online questionnaire survey was conducted among parents of affected children who visited the Department of Pediatrics at Tokyo Rosai Hospital, Rokugo Kodomo Clinic, or Kotokoto Clinic. The survey began on Apr 14, 2022, Aug 8, 2022, and Aug 19, 2022, at the Department of Pediatrics of Tokyo Rosai Hospital, Kotokoto Clinic, and Rokugo Kodomo Clinic, respectively, and was completed at all medical facilities on Feb 4, 2024. A physician distributed a booklet with the study procedure and sanitised wipes as a reward to parents who visited the clinic. A QR code was printed on the booklet to connect participants to the online questionnaire. Study participants were parents who voluntarily connected to the online questionnaire and provided consent. The consent for the study was obtained by pressing the ‘I agree to participate in the study’ button on the online screen. Only those who pressed this button could proceed to the questionnaire screen. The participants watched one of the three animations and answered the questionnaire before and after viewing. The animations were randomly assigned to participants, and both authors and participants were blinded to the animation assignment. Simple randomisation was used to achieve a 1:1:1 ratio.

### Outcome measures

The intention to use antibiotics appropriately was measured via five items, with a 6-point scale ranging from 1 (strongly disagree) to 6 (strongly agree). Based on the six PMT components (severity, vulnerability, response efficacy, self-efficacy, intrinsic rewards, and response costs) that influence the intention to use antibiotics appropriately, 19 items were evaluated, including four items for response costs and three items for each of the other components [30]; a 6-point scale ranging from 1 (strongly disagree) to 6 (strongly agree) was used. Cronbach's alpha coefficients from previous studies [30] were as follows: intention to use antibiotics appropriately, 0.81; severity, 0.93; vulnerability, 0.94; response efficacy, 0.92; self-efficacy, 0.85; intrinsic rewards, 0.93; and response costs, 0.95. The other items surveyed included knowledge of antibiotics and bacteria (three items), conviction that antibiotics were not prescribed for upper respiratory infection and diarrheal symptoms (two items), experience with inappropriate behaviour related to antibiotic use (four items), and impressions of the animation (four items). An additional questionnaire file shows this in more detail (see Additional file 1). We also collected data on various characteristics, such as sex, age, work pattern, history of antibiotic use, relationship with the affected child, age of the affected child (if there was more than one child, the age of the youngest child was considered), history of antibiotic use for the child, history of hospitalisation for the child, and presence of caregivers when the child was sick. Responses were provided for the following items before and after viewing the animation: intention to use antibiotics appropriately, PMT components that influence the intention to use antibiotics appropriately, knowledge of antibiotics and bacteria, and conviction that antibiotics were not prescribed for upper respiratory infection and diarrheal symptoms.

### Sample size

The sample size was calculated using an effect size of 0.62 for the intention to use antibiotics appropriately, which was calculated by a pre-survey of 25 respondents. Using an effect size of 0.6, α = 0.05, and power of 0.8, the sample size for the Wilcoxon signed-rank test was calculated using G*Power 3.1.9.7 [31, 32], resulting in 25 participants per group. Therefore, a total of 75 participants were required for all groups.

### Statistical analyses

For components such as the intention to use antibiotics appropriately, knowledge of antibiotics and bacteria, and conviction that antibiotics were not prescribed for upper respiratory infection and diarrheal symptoms, scores were calculated as a sum of the scores of each item constituting the component. For the PMT components, the mean score was calculated for each component, and the median score was determined. These analyses were performed using the Wilcoxon signed-rank test before and after viewing the animation in each group. The Kruskal–Wallis test was performed for the analysis of the age of the affected child, and the χ^2^ test or Fisher’s exact test was performed for analysing the other participant characteristics as well as participant experience with inappropriate behaviour related to antibiotic use. The Kruskal–Wallis test was also performed to analyse participant impressions regarding the animations. The significance level was set at *p* < 0.05. The scores for the intention to use antibiotics appropriately, knowledge of antibiotics and bacteria, and conviction that antibiotics were not prescribed for upper respiratory infection and diarrheal symptoms were each calculated as effect sizes. All statistical analyses were performed using IBM SPSS Statistics for Windows (version 27.0; IBM Corp., Armonk, NY, USA).

## Results

**Fig. 1:**
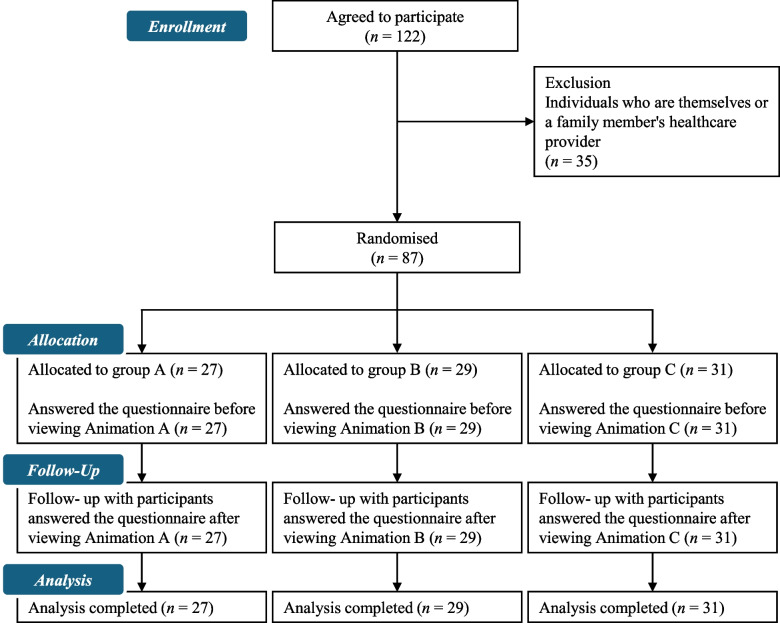
Flow diagram of participant recruitment in this intervention study. This diagram illustrates the participant selection process from enrolment to analysis in this study

The video playback times were 55, 82, and 79 s for animations A, B, and C, respectively. An additional file shows this in more detail (see Additional file 2). A total of 636 booklets were distributed, 122 were accessed via a QR code, 35 were excluded, and 87 responses were received. The collection rate was 13.7%. As a result, responses were received from 27, 29, and 31 participants for animations A, B, and C, respectively.Please check if figures was captured and presented correctly. Figure 1 illustrates the participant recruitment process, from enrolment to analysis, in this study. No participant discontinued answering the questionnaire or viewing the animations during the session. The trial concluded once the required number of samples was obtained. Table 1 summarises the data on various characteristics and responses regarding experience with inappropriate antibiotic use-related behaviour. No significant between-group differences were observed for any item in terms of data on various characteristics and inappropriate antibiotic use-related behaviour.

**Table 1:** Data on various characteristics and responses regarding experience with inappropriate antibiotic use-related behaviour

		Group A	Group B	Group C	*p*
Variable	Category	(*n* = 27)	(*n* = 29)	(*n* = 31)	
Sex: n (%)	Female	22 (81.5)	25 (86.2)	27 (87.1)	0.867^a^
Age: n (%)	20–29 years	0 (0.0)	1 (3.4)	1 (3.2)	0.348^a^
	30–39 years	14 (51.9)	17 (58.6)	13 (41.9)	
	40–49 years	13 (48.1)	11 (37.9)	14 (45.2)	
	50–54 years	0 (0.0)	0 (0.0)	3 (9.7)	
Work pattern: n (%)	Regular employee	13 (48.1)	14 (48.3)	17 (54.8)	0.571^a^
	Independent business	0 (0.0)	1 (3.4)	2 (0.1)	
	Part-time or temporary worker	5 (18.5)	9 (31.0)	7 (22.6)	
	Full-time Homemaker	9 (33.3)	5 (17.2)	5 (16.1)	
Has experience receiving antibiotics: n (%)		26(96.3)	28 (96.6)	29 (93.5)	1.000^a^
Relationships with the patient: n (%)	father or mother	27 (100.0)	29 (100.0)	31 (100.0)	-
Age of patient: Median [IQR]		3.0	5.0	3.0	0.689^b^
		[1.0–8.0]	[2.0–7.5]	[1.0–10.0]	
Has experience of children receiving antibiotics: n (%)		18 (66.7)	19 (65.5)	22 (71.0)	0.892^c^
Has experience of children being hospitalised: n (%)		10 (37.0)	8 (27.6)	10 (32.3)	0.751^c^
Child’s caregivers present: n (%)		23 (85.2)	25 (86.2)	25 (80.6)	0.874^a^
Requested physician to prescribe antibiotics: n (%)	Always	0 (0.0)	0 (0.0)	1 (3.2)	0.564^a^
	Usually	1 (3.7)	2 (6.9)	1 (3.2)	
	Sometimes	1 (3.7)	2 (6.9)	5 (16.1)	
	Never	25 (92.6)	25 (86.2)	24 (77.4)	
Visited another hospital to get antibiotics: n (%)	Always	0 (0.0)	1 (3.4)	0 (0.0)	0.593^a^
	Usually	0 (0.0)	0 (0.0)	1 (3.2)	
	Sometimes	1 (3.7)	0 (0.0)	0 (0.0)	
	Never	26 (96.3)	28 (96.6)	30 (96.8)	
Had taken the leftover antibiotics: n (%)	Always	0 (0.0)	2 (6.9)	0 (0.0)	0.517^a^
	Usually	0 (0.0)	1 (3.4)	2 (6.5)	
	Sometimes	2 (7.4)	1 (3.4)	3 (9.7)	
	Never	25 (92.6)	25 (86.2)	26 (83.9)	
Stopped taking antibiotics at the parent’s discretion: n (%)	Always	0 (0.0)	1 (3.4)	1 (3.2)	0.628^a^
	Usually	4 (14.8)	3 (10.3)	4 (12.9)	
	Sometimes	8 (29.6)	5 (17.2)	11 (35.5)	
	Never	15 (55.6)	20 (69.0)	15 (48.4)	

*IQR* interquartile range

^a^Fisher’s exact test

^b^Kruskal–Wallis test

^c^χ2 test

Table 2 shows the results of the following components: intention to use antibiotics appropriately, evaluation items based on the PMT, knowledge of antibiotics and bacteria, and the conviction that antibiotics were not prescribed for upper respiratory infection and diarrheal symptoms. The median [interquartile range, IQR] score of the intention to use antibiotics appropriately increased significantly from 27.0 [24.0–29.0] to 30.0 [28.0–30.0] in group A and 27.0 [24.0–29.0] to 30.0 [26.0–30.0] in group C (*p* = 0.001, *p* < 0.001). Both groups showed large effect sizes (*r* = 0.62 [group A] and *r* = 0.64 [group C]) [33]. In group B, the score increased from 27.0 [25.0–27.0] to 28.0 [25.0–30.0], which was not significant (*p* = 0.237). Regarding the evaluation items based on the PMT, the severity score increased significantly from 4.0 [3.7–4.7] to 5.0 [4.7–6.0] in group A and 4.0 [3.7–5.0] to 6.0 [5.0–6.0] in group C (*p* < 0.001, *p* = 0.001), with both groups showing large effect sizes (*r* = 0.76 [group A] and *r* = 0.60 [group C]). In group B, the score increased from 4.0 [3.0–4.7] to 4.7 [2.5–5.8], which was not significant (*p* = 0.589). The response costs score decreased significantly from 2.0 [1.3–3.0] to 1.0 [1.0–2.5] in group A and 2.0 [1.0–2.8] to 2.0 [1.0–2.0] in group C (*p* = 0.001, *p* = 0.022), with a large effect size for group A (*r* = 0.62) and a moderate effect size for group C (*r* = 0.41). In group B, the score decreased from 2.0 [1.1–2.1] to 1.3 [1.0–2.0], which was not significant (*p* = 0.167). The median [IQR] score of knowledge of antibiotics and bacteria increased significantly from 11.0 [0.0–13.0] to 14.0 [13.0–16.0], 12.0 [11.0–14.5] to 13.0 [11.0–17.0], and 12.0 [10.0–15.0] to 15.0 [11.0–17.0] in groups A (*p* < 0.001), B (*p* = 0.046), and C (*p* = 0.010), respectively. The effect size was large for group A (*r* = 0.71) and moderate for groups B (*r* = 0.37) and C (*r* = 0.46). The median [IQR] score for the conviction that antibiotics were not prescribed for upper respiratory infection and diarrheal symptoms increased significantly from 10.0 [8.0–11.0] to 12.0 [10.0–12.0], 10.0 [10.0–10.5] to 12.0 [10.0–12.0], and 10.0 [8.0–11.0] to 12.0 [10.0–12.0] in groups A (*p* = 0.002), B (*p* = 0.027), and C (*p* = 0.005), respectively. The effect sizes were large for groups A (*r* = 0.60) and C (*r* = 0.51) and moderate for group B (*r* = 0.41).

**Table 2:** Outcome of educational animations

	Group A				Group B				Group C			
	(*n* = 27)				(*n* = 29)				(*n* = 31)			
	Before	After	*p*	*r*	Before	After	*p*	*r*	Before	After	*p*	*r*
Intention to use antibiotics appropriately: Median [IQR]	27.0 [24.0–29.0]	30.0 [28.0–30.0]	0.001	0.62	27.0 [25.0–27.0]	28.0 [25.0–30.0]	0.237	0.22	27.0 [24.0–29.0]	30.0 [26.0–30.0]	< 0.001	0.64
Protection motivation theory constructs: Median [IQR]												
Severity	4.0 [3.7–4.7]	5.0 [4.7–6.0]	< 0.001	0.76	4.0 [3.0–4.7]	4.7 [2.5–5.8]	0.589	0.10	4.0 [3.7–5.0]	6.0 [5.0–6.0]	0.001	0.60
Vulnerability	3.7 [3.0–4.7]	5.3 [5.0–6.0]	< 0.001	0.78	4.0 [3.0–5.0]	5.0 [4.0–6.0]	0.005	0.52	4.3 [3.3–5.0]	6.0 [5.0–6.0]	< 0.001	0.64
Response efficacy	4.0 [3.7–5.0]	5.3 [5.0–6.0]	< 0.001	0.83	4.0 [3.0–4.7]	5.0 [4.0–6.0]	< 0.001	0.66	4.0 [3.3–5.0]	5.0 [5.0–6.0]	< 0.001	0.68
Self-efficacy	5.0 [4.0–6.0]	6.0 [5.0–6.0]	0.003	0.58	5.0 [4.0–6.0]	5.7 [5.0–6.0]	0.028	0.41	5.0 [4.0–6.0]	6.0 [5.0–6.0]	0.002	0.55
Intrinsic rewards	2.0 [1.0–3.0]	1.0 [1.0–2.0]	0.001	0.65	2.0 [1.0–2.5]	1.0 [1.0–2.0]	0.009	0.48	2.0 [1.0–2.7]	1.0 [1.0–2.0]	0.018	0.43
Response costs	2.0 [1.3–3.0]	1.0 [1.0–2.5]	0.001	0.62	2.0 [1.1–2.1]	1.3 [1.0–2.0]	0.167	0.26	2.0 [1.0–2.8]	2.0 [1.0–2.0]	0.022	0.41
Knowledge of antibiotics and bacteria: Median [IQR]	11.0 [10.0–13.0]	14.0 [13.0–16.0]	< 0.001	0.71	12.0 [11.0–14.5]	13.0 [11.0–17.0]	0.046	0.37	12.0 [10.0–15.0]	15.0 [11.0–17.0]	0.010	0.46
Convinced when antibiotics were not prescribed: Median [IQR]	10.0 [8.0–11.0]	12.0 [10.0–12.0]	0.002	0.60	10.0 [10.0–10.5]	12.0 [10.0–12.0]	0.027	0.41	10.0 [8.0–11.0]	12.0 [10.0–12.0]	0.005	0.51

*IQR* interquartile range

Wilcoxon signed-rank test

Table 3 shows the participant impressions regarding the animations. The median [IQR] numbers of responses to the question of whether the animations were easy to understand were 5.0 [5.0–6.0], 5.0 [4.0–6.0], and 5.0 [5.0–6.0] in groups A, B, and C, respectively, with no significant between-group differences (*p* = 0.483). The median [IQR] numbers of responses to the question of whether participants knew the content of the animations were 4.0 [2.0–4.0], 4.0 [2.0–4.5], and 3.0 [2.0–5.0] for groups A, B, and C, respectively, with no significant between-group differences (*p* = 0.952). The median [IQR] number of responses regarding animation speed were 3.0 [3.0–3.0] for group A, 3.0 [3.0–3.0] for group B, and 3.0 [3.0–3.0] for group C, with no significant between-group differences (*p* = 0.468). The median [IQR] number of responses for animation viewing time was 3.0 [3.0–3.0] for groups A, B, or C, indicating a significant difference between the groups (*p* = 0.023). Multiple comparison results showed significant differences between groups A and C and between groups B and C (*p* < 0.018).

**Table 3:** Responses to participant impressions regarding the animations

		Group A		Group B		Group C		*p*
		(*n* = 27)		(*n* = 29)		(*n* = 31)		
Variable	Category	n (%)	Median [IQR]	n (%)	Median [IQR]	n (%)	Median [IQR]	
Was the animation easy to understand?	1. Strongly disagree	0 (0.0)	5.0 [5.0–6.0]	1 (3.4)	5.0 [4.0–6.0]	1 (3.2)	5.0 [5.0–6.0]	0.483
	2. Disagree	1 (3.7)		1 (3.4)		1 (3.2)		
	3. Somewhat disagree	0 (0.0)		1 (3.4)		0 (0.0)		
	4. Somewhat agree	4 (14.8)		7 (24.1)		4 (12.9)		
	5. Agree	11 (40.7)		9 (31.0)		12 (38.7)		
	6. Strongly agree	11 (40.7)		10 (34.5)		13 (41.9)		
Did you know what the animation was about?	1. Strongly disagree	6 (22.2)	4.0 [2.0–4.0]	6 (20.7)	4.0 [2.0–4.5]	6 (19.4)	3.0 [2.0–5.0]	0.952
	2. Disagree	2 (7.4)		4 (13.8)		3 (9.7)		
	3. Somewhat disagree	3 (11.1)		4 (13.8)		8 (25.8)		
	4. Somewhat agree	10 (37.0)		8 (27.6)		6 (19.4)		
	5. Agree	4 (14.8)		4 (13.8)		7 (22.6)		
	6. Strongly agree	2 (7.4)		3 (10.3)		1 (3.2)		
How was the speed of the animation?	1. Fast	0 (0.0)	3.0 [3.0–3.0]	0 (0.0)	3.0 [3.0–3.0]	1 (3.2)	3.0 [3.0–3.0]	0.468
	2. Somewhat fast	5 (18.5)		5 (17.2)		3 (9.7)		
	3. Just right	22 (81.5)		23 (79.3)		24 (77.4)		
	4. Somewhat slow	0 (0.0)		1 (3.4)		3 (9.7)		
	5. Slow	0 (0.0)		0 (0.0)		0 (0.0)		
How did you feel about the animation viewing time?	1. Long	0 (0.0)	3.0 [3.0–3.0]	0 (0.0)	3.0 [3.0–3.0]	0 (0.0)	3.0 [3.0–3.0]	0.023
	2. Somewhat long	0 (0.0)		1 (3.4)		5 (16.1)		
	3. Just right	26 (96.3)		26 (89.7)		26 (83.9)		
	4. Somewhat short	1 (3.7)		2 (6.9)		0 (0.0)		
	5. Short	0 (0.0)		0 (0.0)		0 (0.0)		

Kruskal–Wallis test

*IQR* interquartile range

## Discussion

In this study, we used behavioural economics to educate Japanese parents about the appropriate use of antibiotics. After watching animations lacking message framing, significant differences were observed across all assessed items, including scores for the intention to use antibiotics appropriately, knowledge of antibiotics and bacteria, and conviction that no antibiotics prescribed for upper respiratory infection and diarrheal symptoms. Furthermore, these effect sizes of the animation were substantial. Specifically, scores for knowledge of antibiotics and bacteria increased significantly after watching all animations, with the animation without message framing showing the largest effect size. Importantly, this animation also had the shortest duration. It must be noted that the survey was conducted among parents of sick children, potentially limiting the time available to watch animations. We believe that shorter animations can positively influence their effectiveness, particularly for parents of sick children. Thus, animations with concise and clear messages are deemed effective for this audience. However, while the provision of one-sided information improves knowledge, once a certain threshold is reached, such information is no longer effective in further increasing the level of knowledge [34]. In addition to a lack of knowledge, expectations regarding antibiotics, driven by past experiences and a desire for antibiotic prescription, have been reported to contribute to parental concerns [13]. Especially in Japan, where parents must balance work and childcare, there is a tendency to rely on antibiotics. The animation incorporating loss-framing messages has a smaller effect on improving knowledge compared to this animation; however, it exerts similar influence on intentions to use antibiotics appropriately. Therefore, we suggest that using animations with loss-framing messages, rather than relying solely on one-way information provision, may effectively enhance the intention to use antibiotics appropriately, even without significantly increasing knowledge.

Comparing animations with additional message framing showed a significant improvement in participant intention to use antibiotics appropriately after watching animations with loss-framing messages; however, the difference was not significant after watching animations with gain-framing messages. This indicates that the addition of gain-framing messages may not be effective in educating parents about the appropriate use of antibiotics. Reportedly, the effectiveness of message framing depends on an individual’s perception of the risk associated with health behaviours, and the use of loss-framing messages is considered effective when the risk is high [35]. In this study, the addition of a loss-framing message improved participant intention to use antibiotics appropriately, suggesting that parents perceived the possibility of an increase in antimicrobial resistance due to the unnecessary use of antibiotics as a health risk.

The addition of gain-framing messages did not significantly change PMT components, such as severity and response costs. Severity is a factor that represents the perception of risk due to antimicrobial resistance, thereby improving the intention to use antibiotics appropriately [28]. It is suggested that positive expressions reduce the awareness of the risk severity of antimicrobial resistance and, hence, do not improve the intention to use antibiotics appropriately. By contrast, response costs represent the perception of the burden associated with the appropriate use of antibiotics, and a decrease in response costs enhances the intention to use antibiotics appropriately [28]. The gain-framing message emphasised the benefits of using antibiotics because it depicted that children’s illnesses improved with the use of antibiotics. Therefore, we believe that response costs were not affected. In a previous study investigating the relationship between PMT components and antibiotic adherence, response costs were most strongly correlated with adherence [36]. The findings of the present study are consistent with those of the previous one, as only the gain-framing messages, which did not significantly reduce response costs, failed to increase the intention to use antibiotics appropriately. In addition to PMT [28], self-efficacy is recognised as a factor influencing health behaviours in both the social cognition model [37] and the health belief model [38]. Self-efficacy increased significantly before and after viewing both animations; however, the loss-framing message yielded a larger effect size than did the gain-framing, suggesting a potentially greater influence on health behaviours.

Previous studies have reported that loss-framing messages are more effective than gain-framing messages in terms of enhancing nutrition-related self-management for gastric cancer patients and adherence to treatments for patients with obstructive sleep apnoea [39, 40]. The loss-framing messages proved more successful in these previous studies because the focus was on the patients themselves. It is plausible that the loss-framing was also effective in this study, targeting parents who were closely involved with their patients.

After watching animations, both gain- and loss-framing messages resulted in significantly higher scores for participant conviction that antibiotics were not prescribed for upper respiratory infection and diarrheal symptoms; however, the effect size was greater for loss-framing animations than for gain-framing animations. In a previous survey of doctors across Japan, more than half reported they would prescribe antibiotics even after explaining to patients their　initial decision against prescribing them if they were not convinced[41]. This lack of conviction in not prescribing antibiotics for symptoms of upper respiratory infection and diarrhoea could foster demand for antibiotics. Therefore, employing loss-framing animations might strengthen conviction, thus reducing unnecessary antibiotic use. Moreover, using these animations instead of a doctor’s explanation could serve as an effective tool for busy doctors to promote appropriate antibiotic use. In addition, many participants believed that the viewing time for loss-framing animation was longer than that for gain-framing animations or animations that did not use message framing. Therefore, we believe that the negative atmosphere created by loss-framing messages gave viewers the impression that the viewing time was longer.

As no previous study has examined the impact of message framing on the intention to use antibiotics appropriately, this research was conducted without considering personal factors such as parents' concerns about their children’s illnesses. However, our previous study [13] found that mothers who were unconvinced by the absence of an antibiotic prescription had positive prior experiences with antibiotics and were particularly concerned about their children's illnesses. Therefore, we intend to assess the effect of the animations on parents with such personal factors in future research.

### Limitations

This study has some limitations. First, as participants in this study were recruited on a voluntary basis, it is possible that many had a prior interest in antibiotics and antimicrobial resistance. However, we believe that potential confounding factors were mitigated to some extent through the random assignment of participants to the different animation groups. Moreover, a pre-intervention survey revealed no significant differences among the three groups in terms of their perception of the animation content or their baseline knowledge regarding the function of antibiotics and antimicrobial resistance. Notably, most participants completed the survey at home rather than at the medical institutions where they received the survey booklet, allowing them to respond without concern for the opinion of medical professionals. Second, the data were collected from a few local medical institutions, which may limit the generalizability of findings. In addition, because the study targeted parents of affected children, participation may have been low due to nursing care. In future research, we aim to conduct a nationwide survey in nurseries or online settings that would allow parents to view animations in a calm environment, free from conflicts of interest related to antibiotic use and antimicrobial resistance. Lastly, the effectiveness of animations was measured immediately after viewing; therefore, it was unclear whether the effect was long-lasting. Additionally, because this study measured behavioural intentions, it could not investigate actual behavioural changes. Future research should examine the long-term impact of animations, including actual behavioural change. However, the observed shifts in PMT components [26, 27], which are known to affect health behaviour, support the value of this outcome in demonstrating the effectiveness of the educational animation in promoting appropriate antibiotic use among parents.

## Conclusions

The study suggests that incorporating a loss-framing message can effectively enhance parents' intentions to use antibiotics appropriately as part of an animation scenario. The gain-framing message, which highlighted the benefits of antibiotics and downplayed the risks associated with their unnecessary use, did not appear to improve the intention to use antibiotics appropriately. Therefore, when supplementing knowledge about antimicrobials, it is not recommended to use gain-framing messages. Instead, content conveying the disadvantages of unnecessary antibiotic use to parents may be more effective, and the loss-framing message is appropriate.

## Supplementary Information

Additional file 1. Questionnaire. This additional file includes the questionnaire used in this study before and after watching the animation.

Additional file 2. Storyline of the animations. Description of data: This additional file contains details of the storyline of the animations created for the study.

Additional file 3. CONSORT 2010 checklist of information to include when reporting a randomised trial. This additional file contains CONSORT 2010 Checklist with reported on page numbers.

## Data Availability

The datasets used and/or analysed during the current study are available from the corresponding author upon reasonable request.

## Abbreviations

PMT: Protection motivation theory
IBM: International Business Machines Corporation
IQR: Interquartile Range
SPSS: Statistical Package for the Social Sciences
