# Supplementary material for: Effects of educational animations using message framing on the appropriate antibiotic use by parents: a randomised, three-armed intervention study
Source: BMC Public Health. 2025 Jul 3;25:2360. doi: 10.1186/s12889-025-23577-4 (PMC12224803; doi:10.1186/s12889-025-23577-4)
Supplement: Supplementary file 1 — Additional file 1. Questionnaire. This additional file includes the questionnaire used in this study before and after watching the animation. [file 12889_2025_23577_MOESM1_ESM.docx]

**Additional file 1. Questionnaire**

[Pre-animation viewing]

Q1. Please respond to the following questions.

|  |  | Strongly disagree | Disagree | Somewhat disagree | Somewhat agree | Agree | Strongly agree |
| --- | --- | --- | --- | --- | --- | --- | --- |
| 1 | Do you think most colds are caused by viruses? | 1 | 2 | 3 | 4 | 5 | 6 |
| 2 | Do you think antibiotics work for infections caused by viruses? | 1 | 2 | 3 | 4 | 5 | 6 |
| 3 | Do you think bacteria and viruses are the same? | 1 | 2 | 3 | 4 | 5 | 6 |

Q2. Please share your personal experience. **Kindly include your child’s experience in your answer.**

|  |  | Always | Usually | Sometimes | Never |
| --- | --- | --- | --- | --- | --- |
| 1 | **I requested antibiotics and asked the physician to prescribe them** because they were not prescribed. | 1 | 2 | 3 | 4 |
| 2 | I requested antibiotics, **but they were not prescribed; hence, I visited other hospitals to have them prescribed.** | 1 | 2 | 3 | 4 |
| 3 | I requested antibiotics, **but they were not prescribed; hence, I used antibiotics that were already at home.** | 1 | 2 | 3 | 4 |
| 4 | **I stopped taking** a prescribed antibiotic at my own discretion without completing the full course, or **I stopped giving it to my child.** | 1 | 2 | 3 | 4 |

Q3. Please answer the following questions regarding what you think would happen **to your child.**

|  |  | Strongly disagree | Disagree | Somewhat disagree | Somewhat agree | Agree | Strongly agree |
| --- | --- | --- | --- | --- | --- | --- | --- |
| 1 | A physician told you that antibiotics are not necessary for **any of the following symptoms: fever, runny nose, nasal congestion, sore throat, cough, or phlegm.** Can you accept that antibiotics are not prescribed? | 1 | 2 | 3 | 4 | 5 | 6 |
| 2 | A physician told you that antibiotics are not necessary for diarrhoea. Can you accept that antibiotics are not prescribed? | 1 | 2 | 3 | 4 | 5 | 6 |

Q4. Please answer the following questions regarding what you think would happen **to your child.**

|  |  | Strongly disagree | Disagree | Somewhat disagree | Somewhat agree | Agree | Strongly agree |
| --- | --- | --- | --- | --- | --- | --- | --- |
| 1 | If the symptoms go away, do you stop giving your child the prescribed antibiotics at your own discretion without finishing them? (If your child has never been prescribed antibiotics, assume they have.) | 1 | 2 | 3 | 4 | 5 | 6 |
| 2 | If you visited a health care provider because your child had a fever, sniffle, congestion, sore throat, cough, or phlegm, but the doctor told you that your child did not need antibiotics, would you ask for a prescription for antibiotics? | 1 | 2 | 3 | 4 | 5 | 6 |
| 3 | If you visited a health care provider because your child had a fever, sniffle, congestion, sore throat, cough, or phlegm, but the doctor told you that your child did not need antibiotics, would you use your own judgement in using antibiotics left at home? | 1 | 2 | 3 | 4 | 5 | 6 |
| 4 | If you visited a health care provider because your child had diarrhoea, but the doctor told you that your child did not need antibiotics, would you ask for a prescription for antibiotics? | 1 | 2 | 3 | 4 | 5 | 6 |
| 5 | If you visited a health care provider because your child had diarrhoea, but the doctor told you that your child did not need antibiotics, would you use your own judgement in using antibiotics left at home? | 1 | 2 | 3 | 4 | 5 | 6 |

Q5. Please answer the following questions regarding what you think would happen **to your child.**

|  |  | Strongly disagree | Disagree | Somewhat disagree | Somewhat agree | Agree | Strongly agree |
| --- | --- | --- | --- | --- | --- | --- | --- |
| Severity | | | | | | | |
| 1 | Antimicrobial resistance can render disease symptoms more severe. | 1 | 2 | 3 | 4 | 5 | 6 |
| 2 | Antimicrobial resistance may prevent the successful treatment of various infections. | 1 | 2 | 3 | 4 | 5 | 6 |
| 3 | Antimicrobial resistance may reduce the number of antibiotics that can be used in the future. | 1 | 2 | 3 | 4 | 5 | 6 |

Q6. Please answer the following questions, assuming a situation in which **your child will use antibiotics.**

|  |  | Strongly disagree | Disagree | Somewhat disagree | Somewhat agree | Agree | Strongly agree |
| --- | --- | --- | --- | --- | --- | --- | --- |
| Vulnerability | | | | | | | |
| 1 | The unnecessary use of antibiotics increases the likelihood that your variety of infections will not be successfully treated. | 1 | 2 | 3 | 4 | 5 | 6 |
| 2 | The unnecessary use of antibiotics increases the likelihood that your infection will become more severe. | 1 | 2 | 3 | 4 | 5 | 6 |
| 3 | The unnecessary use of antimicrobials increases the likelihood that fewer antibiotics will be available to you in the future. | 1 | 2 | 3 | 4 | 5 | 6 |
| Response efficacy | | | | | | | |
| 4 | The treatment of various infections can be accomplished if you do not demand antibiotics when they are not prescribed. | 1 | 2 | 3 | 4 | 5 | 6 |
| 5 | The severity of the infection can be prevented if you do not demand antibiotics when they are not prescribed. | 1 | 2 | 3 | 4 | 5 | 6 |
| 6 | More antibiotics will be available in the future if you do not demand antibiotics when they are not prescribed. | 1 | 2 | 3 | 4 | 5 | 6 |
| Self-efficacy | | | | | | | |
| 7 | You could hold back from visiting another hospital to get a prescription for antibiotics if you were not prescribed them. | 1 | 2 | 3 | 4 | 5 | 6 |
| 8 | You can choose not to tell your doctor that you want him or her to prescribe antibiotics if you were not prescribed them. | 1 | 2 | 3 | 4 | 5 | 6 |
| 9 | You can choose not to use your extra antibiotics if you were not prescribed them. | 1 | 2 | 3 | 4 | 5 | 6 |
| Intrinsic rewards | | | | | | | |
| 10 | You feel more comfortable using an antibiotic, even if your doctor says they are not necessary. | 1 | 2 | 3 | 4 | 5 | 6 |
| 11 | You are more satisfied with the use of antibiotics, even if your doctor says they are unnecessary. | 1 | 2 | 3 | 4 | 5 | 6 |
| 12 | You trust antibiotics more than fever reducers, cough medicines, etc., even if your doctor says they are unnecessary. | 1 | 2 | 3 | 4 | 5 | 6 |
| Response costs | | | | | | | |
| 13 | You are concerned that you will become seriously ill by not using an antibiotic, even if your doctor says they are unnecessary. | 1 | 2 | 3 | 4 | 5 | 6 |
| 14 | You are concerned that you will prolong your illness by not using an antibiotic, even if your doctor says they are unnecessary. | 1 | 2 | 3 | 4 | 5 | 6 |
| 15 | Even if the doctor says antibiotics are unnecessary, not using them will interfere with your daily life. | 1 | 2 | 3 | 4 | 5 | 6 |
| 16 | Even if the doctor says antibiotics are unnecessary, not using them increases the probability of a return visit to the doctor. | 1 | 2 | 3 | 4 | 5 | 6 |

[Post-animation viewing]

Q7. Please provide your own responses to the following questions.

|  |  | Strongly disagree | Disagree | Somewhat disagree | Somewhat agree | Agree | Strongly agree |
| --- | --- | --- | --- | --- | --- | --- | --- |
| 1 | Do you think most colds are caused by viruses? | 1 | 2 | 3 | 4 | 5 | 6 |
| 2 | Do you think antibiotics work for infections caused by viruses? | 1 | 2 | 3 | 4 | 5 | 6 |
| 3 | Do you think bacteria and viruses are the same? | 1 | 2 | 3 | 4 | 5 | 6 |

Q8. Please answer the following questions regarding what you think would happen **to your child.**

|  |  | Strongly disagree | Disagree | Somewhat disagree | Somewhat agree | Agree | Strongly agree |
| --- | --- | --- | --- | --- | --- | --- | --- |
| 1 | A physician told you that antibiotics are not necessary for **any of the following symptoms: fever, runny nose, nasal congestion, sore throat, cough, or phlegm.** Can you accept that antibiotics are not prescribed? | 1 | 2 | 3 | 4 | 5 | 6 |
| 2 | A physician told you that antibiotics are not necessary for diarrhoea. Can you accept that antibiotics are not prescribed? | 1 | 2 | 3 | 4 | 5 | 6 |

Q9. Please answer the following questions regarding what you think would happen **to your child.**

|  |  | Strongly disagree | Disagree | Somewhat disagree | Somewhat agree | Agree | Strongly agree |
| --- | --- | --- | --- | --- | --- | --- | --- |
| 1 | If the symptoms go away, do you stop giving your child the prescribed antibiotics at your own discretion without finishing them? (If your child has never been prescribed antibiotics, assume they have.) | 1 | 2 | 3 | 4 | 5 | 6 |
| 2 | If you visited a healthcare provider because your child had a fever, sniffle, congestion, sore throat, cough, or phlegm, but the doctor told you that your child did not need antibiotics, would you ask for a prescription for antibiotics? | 1 | 2 | 3 | 4 | 5 | 6 |
| 3 | If you visited a health care provider because your child had a fever, sniffle, congestion, sore throat, cough, or phlegm, but the doctor told you that your child did not need antibiotics, would you use your own judgement in using antibiotics left at home? | 1 | 2 | 3 | 4 | 5 | 6 |
| 4 | If you visited a healthcare provider because your child had diarrhoea, but the doctor told you that your child did not need antibiotics, would you ask for a prescription for antibiotics? | 1 | 2 | 3 | 4 | 5 | 6 |
| 5 | If you visited a healthcare provider because your child had diarrhoea, but the doctor told you that your child did not need antibiotics, would you use your own judgement in using antibiotics left at home? | 1 | 2 | 3 | 4 | 5 | 6 |

Q10. Please answer the following questions regarding what you think would happen **to your child.**

|  |  | Strongly disagree | Disagree | Somewhat disagree | Somewhat agree | Agree | Strongly agree |
| --- | --- | --- | --- | --- | --- | --- | --- |
| Severity | | | | | | | |
| 1 | Antimicrobial resistance can render disease symptoms more severe. | 1 | 2 | 3 | 4 | 5 | 6 |
| 2 | Antimicrobial resistance may prevent the successful treatment of various infections. | 1 | 2 | 3 | 4 | 5 | 6 |
| 3 | Antimicrobial resistance may reduce the number of antibiotics that can be used in the future. | 1 | 2 | 3 | 4 | 5 | 6 |

Q11. Please answer the following questions, assuming a situation in which **your child will use antibiotics.**

|  |  | Strongly disagree | Disagree | Somewhat disagree | Somewhat agree | Agree | Strongly agree |
| --- | --- | --- | --- | --- | --- | --- | --- |
| Vulnerability | | | | | | | |
| 1 | The unnecessary use of antibiotics increases the likelihood that your variety of infections will not be successfully treated. | 1 | 2 | 3 | 4 | 5 | 6 |
| 2 | The unnecessary use of antibiotics increases the likelihood that your infection will become more severe. | 1 | 2 | 3 | 4 | 5 | 6 |
| 3 | The unnecessary use of antimicrobials increases the likelihood that fewer antibiotics will be available to you in the future. | 1 | 2 | 3 | 4 | 5 | 6 |
| Response efficacy | | | | | | | |
| 4 | The treatment of various infections can be accomplished if you do not demand antibiotics when they are not prescribed. | 1 | 2 | 3 | 4 | 5 | 6 |
| 5 | The severity of the infection can be prevented if you do not demand antibiotics when they are not prescribed. | 1 | 2 | 3 | 4 | 5 | 6 |
| 6 | More antibiotics will be available in the future if you do not demand antibiotics when they are not prescribed. | 1 | 2 | 3 | 4 | 5 | 6 |
| Self-efficacy | | | | | | | |
| 7 | You could hold back from visiting another hospital to get a prescription for antibiotics if you were not prescribed them. | 1 | 2 | 3 | 4 | 5 | 6 |
| 8 | You can choose not to tell your doctor that you want him or her to prescribe antibiotics if you were not prescribed them. | 1 | 2 | 3 | 4 | 5 | 6 |
| 9 | You can choose not to use your extra antibiotics if you were not prescribed them. | 1 | 2 | 3 | 4 | 5 | 6 |
| Intrinsic rewards | | | | | | | |
| 10 | You feel more comfortable using an antibiotic, even if your doctor says they are not necessary. | 1 | 2 | 3 | 4 | 5 | 6 |
| 11 | You are more satisfied with the use of antibiotics, even if your doctor says they are unnecessary. | 1 | 2 | 3 | 4 | 5 | 6 |
| 12 | You trust antibiotics more than fever reducers, cough medicines, etc., even if your doctor says they are unnecessary. | 1 | 2 | 3 | 4 | 5 | 6 |
| Response costs | | | | | | | |
| 13 | You are concerned that you will become seriously ill by not using an antibiotic, even if your doctor says they are unnecessary. | 1 | 2 | 3 | 4 | 5 | 6 |
| 14 | You are concerned that you will prolong your illness by not using an antibiotic, even if your doctor says they are unnecessary. | 1 | 2 | 3 | 4 | 5 | 6 |
| 15 | Even if the doctor says antibiotics are unnecessary, not using them will interfere with your daily life. | 1 | 2 | 3 | 4 | 5 | 6 |
| 16 | Even if the doctor says antibiotics are unnecessary, not using them increases the probability of a return visit to the doctor. | 1 | 2 | 3 | 4 | 5 | 6 |

Q12. Please answer the following questions about the animation content.

|  |  | Strongly disagree | Disagree | Somewhat disagree | Somewhat agree | Agree | Strongly agree |
| --- | --- | --- | --- | --- | --- | --- | --- |
| 1 | Was the animation easy to understand? | 1 | 2 | 3 | 4 | 5 | 6 |
| 2 | Did you know what the animation was about? | 1 | 2 | 3 | 4 | 5 | 6 |

Q13. How can you describe the speed of the animation?

　　1. Fast 2. Somewhat fast 3. Just right 4. Somewhat slow 5. Slow

Q14. How did you feel about the animation viewing time?

　　1. Long 2. Somewhat long 3. Just right 4. Somewhat short 5. Short
