# Supplementary material for: Effects of educational animations using message framing on the appropriate antibiotic use by parents: a randomised, three-armed intervention study
Source: BMC Public Health. 2025 Jul 3;25:2360. doi: 10.1186/s12889-025-23577-4 (PMC12224803; doi:10.1186/s12889-025-23577-4)
Supplement: Supplementary file 2 — Additional file 2. Storyline of the animations. Description of data: This additional file contains details of the storyline of the animations created for the study. [file 12889_2025_23577_MOESM2_ESM.docx]

**Additional file 2. Storyline of the animations**

**[Common introduction to all animations A–C]**

| 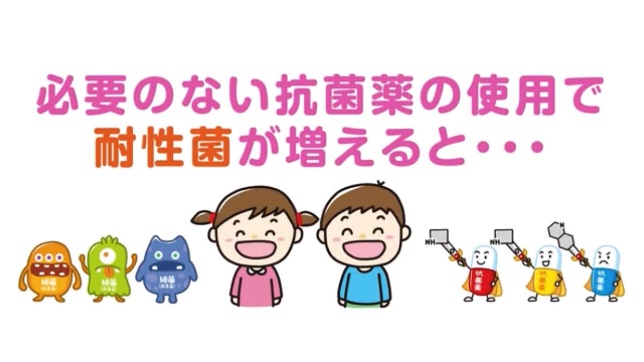①  Unnecessary use of antibiotics increases the number of antimicrobial resistances... | 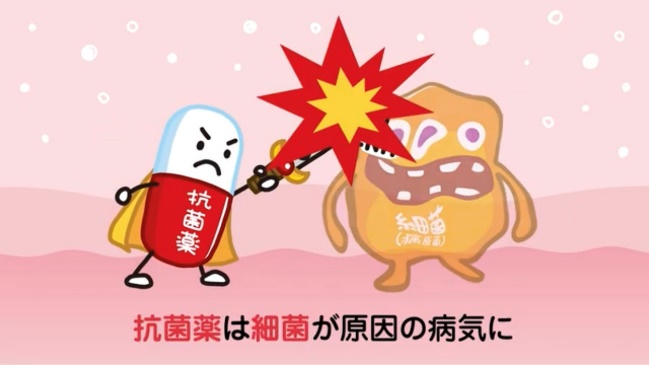②  No antimicrobial resistance  Antibiotic  Antibiotics are effective |
| --- | --- |
| 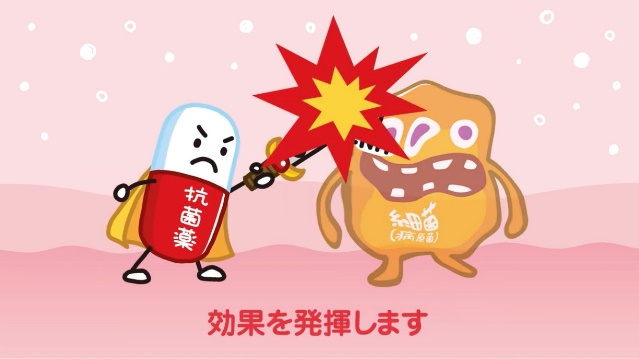③  against diseases caused by bacteria. | ④ 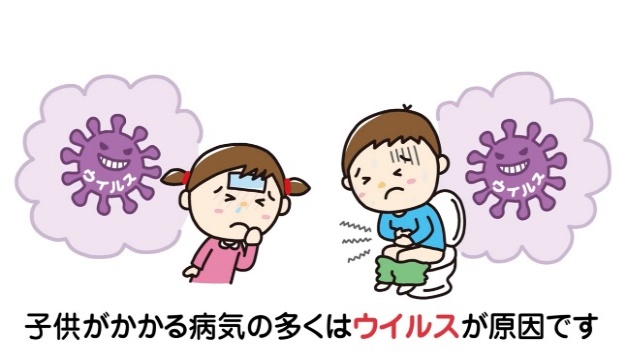 Most diseases in children are caused by viruses.  Virus  Virus |
| 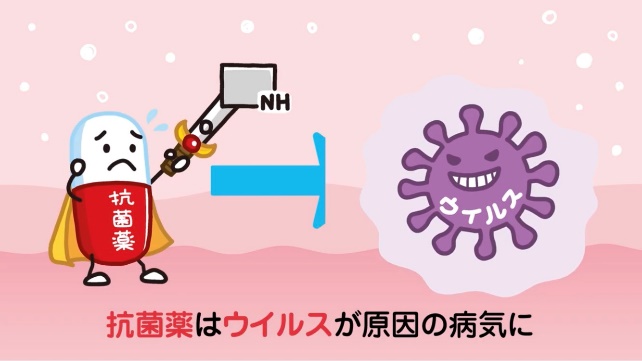⑤  Antibiotics are not necessary | 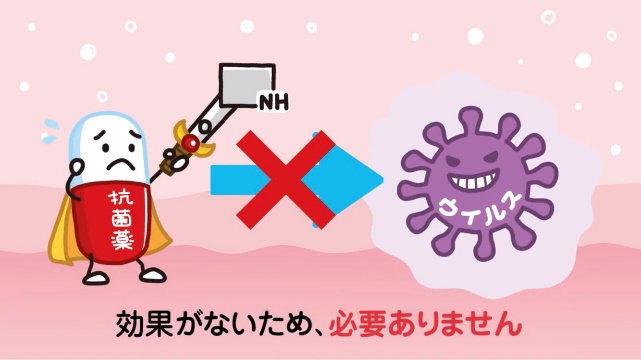⑥  because they are ineffective against diseases caused by viruses. |
| 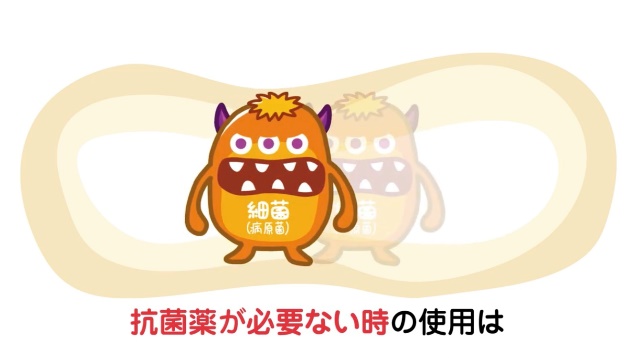⑦  No antimicrobial resistance  Use of antimicrobials | 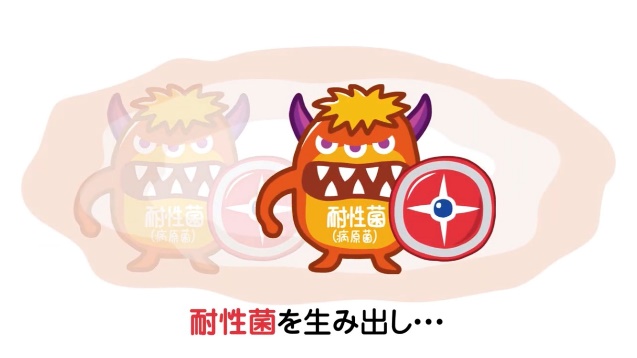⑧  when they are not needed can create  Antimicrobial resistance |
| ⑨ 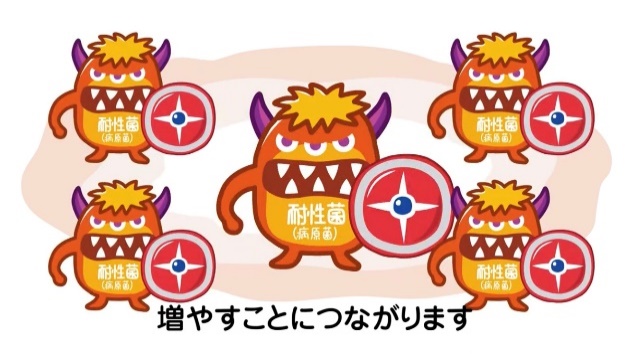 and increase antimicrobial resistance. | 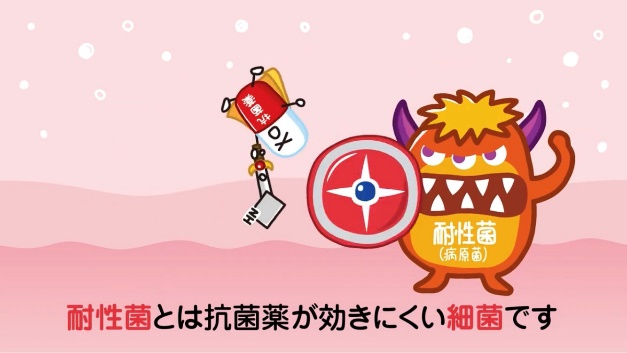⑩  Antimicrobial resistance causes antibiotics to become ineffective. |
| 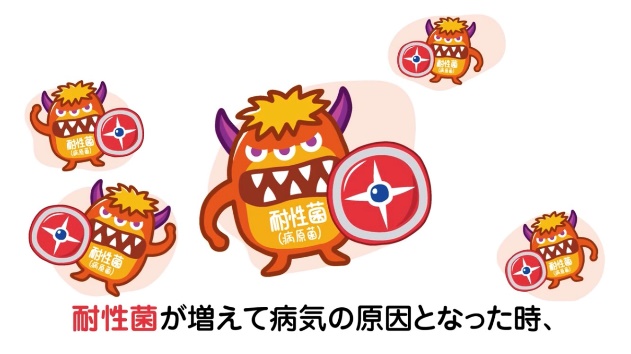⑪  When antimicrobial resistance increases and causes disease, | 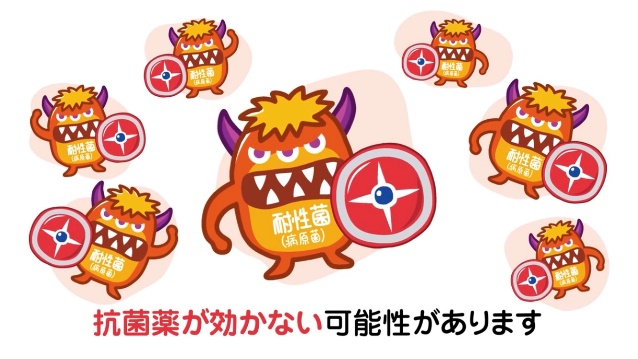⑫  antibiotics may not work. |

**[Animation A]**

**≪After common introduction (①～⑫)≫**

| ⑬ 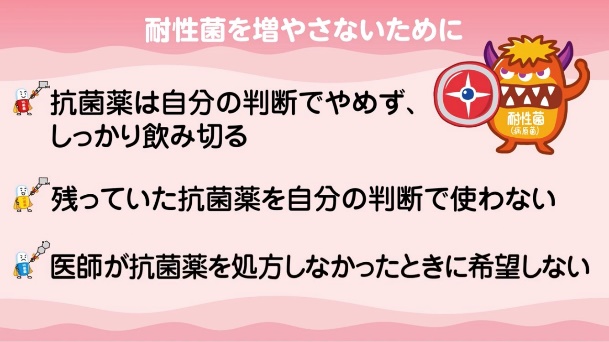 Do not stop antibiotics at your own discretion, but take them as prescribed.  Do not take leftover antibiotics at your own discretion.  Do not ask for antibiotics when the doctor has not prescribed them.  How to not increase antimicrobial resistance | 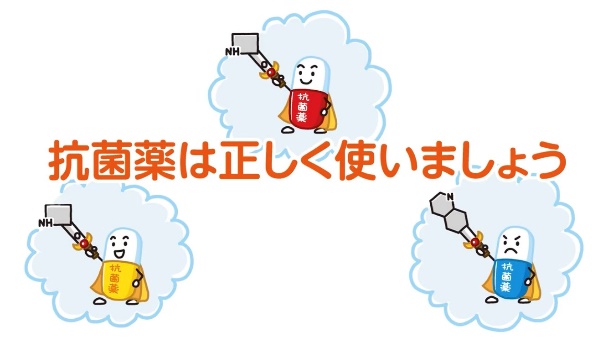⑭  Let's use antibiotics correctly. |
| --- | --- |

**[Animation B]**

**≪After common introduction (①～⑫)≫**

| 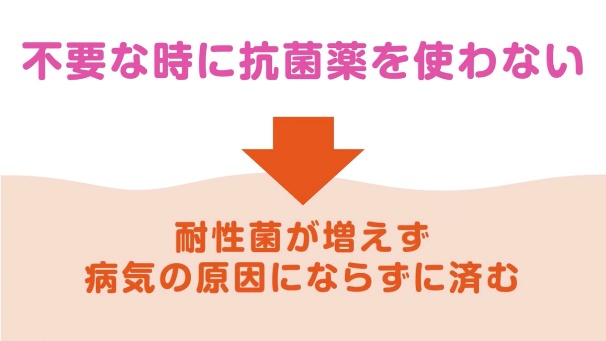⑬  Antimicrobial resistance does not increase and will not become a cause of infectious disease.  Not using antibiotics  when they are not needed. | 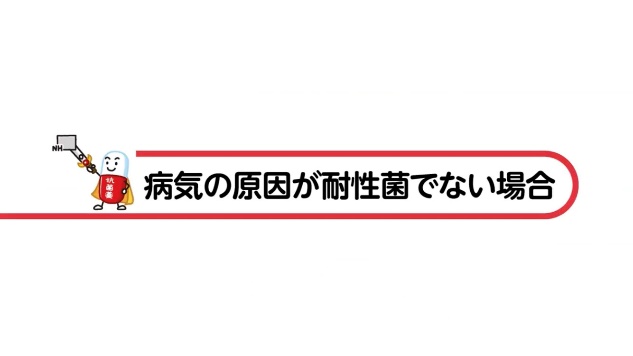⑭  If the cause of the disease is not antimicrobial resistance… |
| --- | --- |
| 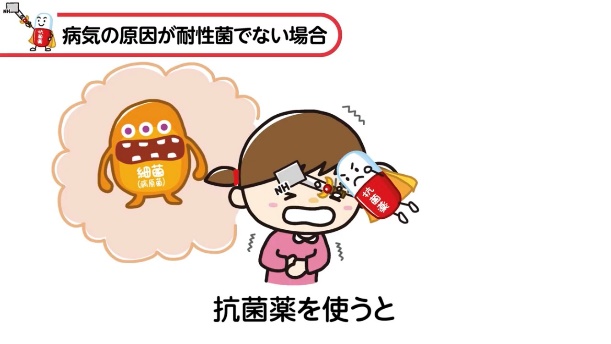⑮  If antibiotics are used,  If the cause of the disease is not antimicrobial resistance… | 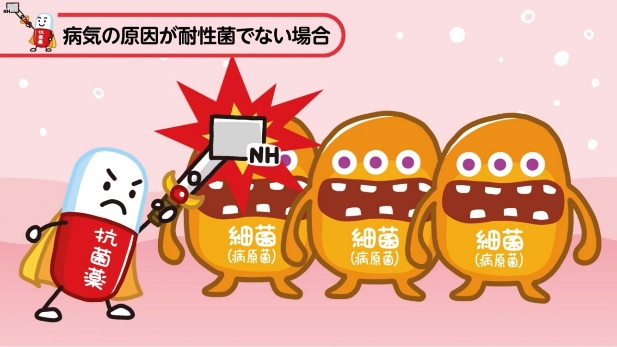⑯  If the cause of the disease is not antimicrobial resistance…  No antimicrobial resistance  Antibiotic |
| 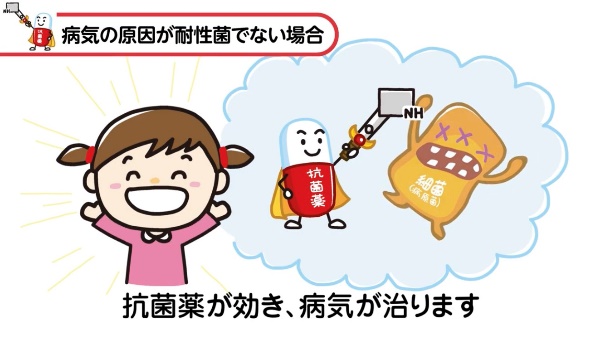⑰  If the cause of the disease is not antimicrobial resistance…  the antibiotics work and the disease is cured. | ⑱ 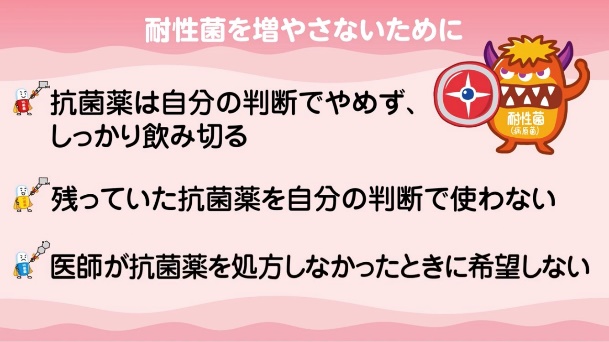 Do not stop antibiotics at your own discretion, but take them as prescribed.  Do not take leftover antibiotics at your own discretion.  Do not ask for antibiotics when the doctor has not prescribed them.  How to not increase antimicrobial resistance |
| ⑲ 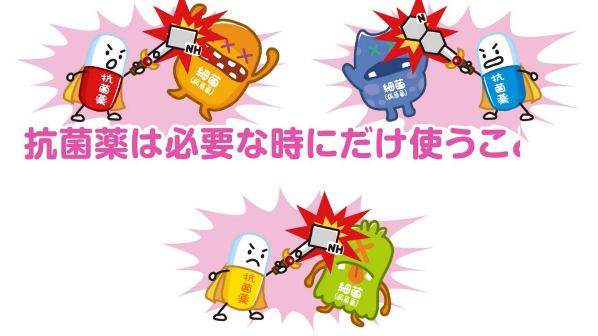 Antibiotics continue to be effective when used only as needed. |  |

**[Animation C]**

**≪After common introduction (①～⑫)≫**

| 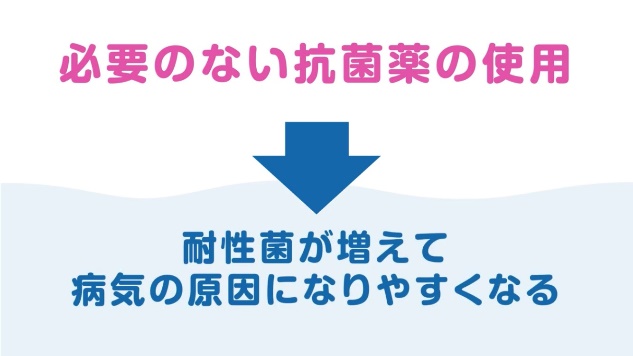⑬  Antimicrobial resistance does increase and will easily become a cause of infectious disease.  Using antibiotics  when they are not needed. | 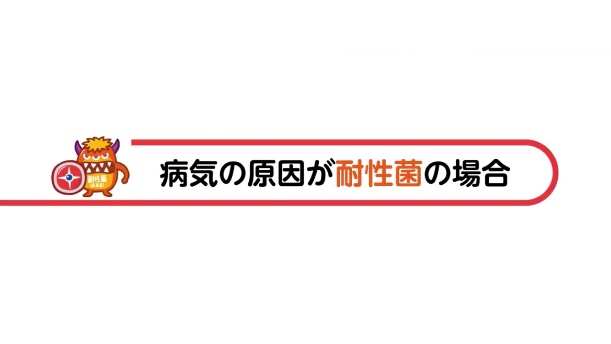⑭  If the cause of the disease is antimicrobial resistance… |
| --- | --- |
| 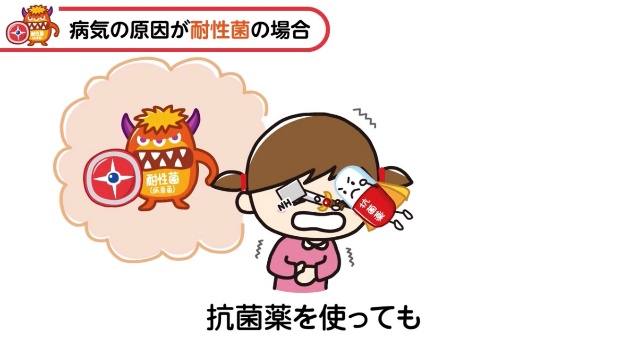⑮  Antibiotic  Antimicrobial  resistance  If antibiotics are used,  If the cause of the disease is antimicrobial resistance… | 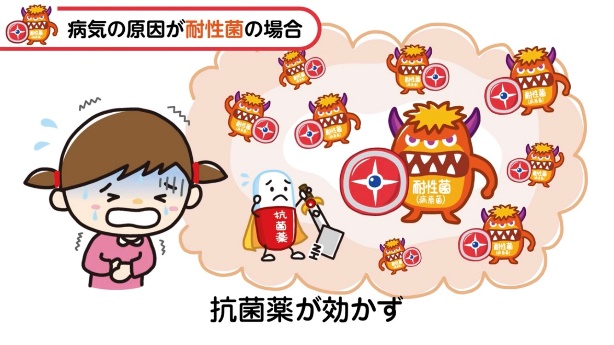⑯  the antibiotics may not work  If the cause of the disease is antimicrobial resistance… |
| 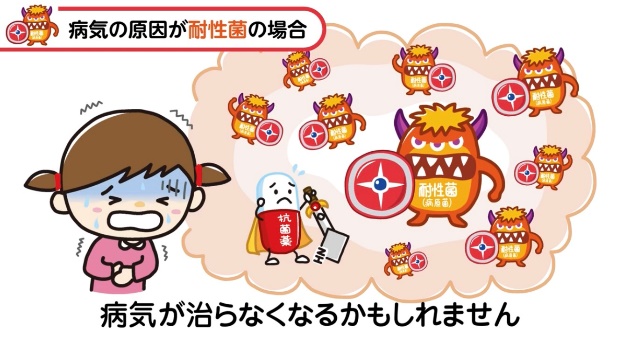⑰  and the disease may not be cured.  If the cause of the disease is antimicrobial resistance… | ⑱ 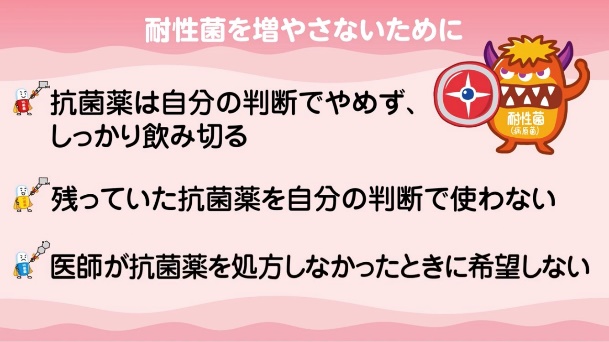 Do not stop antibiotics at your own discretion, but take them as prescribed.  Not taking leftover antibiotics at your own discretion.  Not asking for antibiotics when the doctor has not prescribed them.  How to not increase antimicrobial resistance |
| 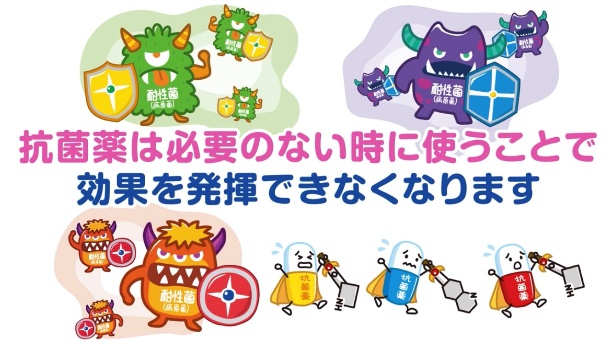⑲  Antibiotics are ineffective  when used unnecessarily. |  |
